# PANoptosis-related gene clusters and prognostic risk model in clear cell renal cell carcinoma

**DOI:** 10.3389/fgene.2025.1605078

**Published:** 2025-11-18

**Authors:** Qiyue Zhao, Huadong Xie, Chaofu Li, Yanxiang Xiong, Yongyi Fan, Yuanbi Huang, Yi Zhan, Siping Zeng

**Affiliations:** 1 Department of Urology, Liuzhou Workers’ Hospital, Liuzhou, Guangxi, China; 2 Department of Oncology, Liuzhou Workers’ Hospital, Liuzhou, Guangxi, China

**Keywords:** PANoptosis, clear cell renal cell carcinoma, prognosis, Tumor microenvironment, drug sensitivity

## Abstract

**Background:**

Despite advancements in targeted therapies, the prognosis for clear cell renal cell carcinoma (ccRCC) remains poor, particularly for metastatic cases. PANoptosis, a newly discovered programmed cell death pathway involving crosstalk among pyroptosis, apoptosis, and necroptosis, has an undefined role in ccRCC pathogenesis and prognosis, representing a critical knowledge gap.

**Methods:**

We conducted a bioinformatics analysis of the expression PANoptosis-related genes (PRGs) in 524 ccRCC patients from the TCGA and GEO databases. Three ccRCC clusters were identified based on PRG expression. Innovatively, we developed a prognostic risk model using LASSO and Cox regression on three hub genes (WDR72, ANLN, SLC16A12), integrating multi-omics data for immune microenvironment, tumor mutation burden (TMB), cancer stem cell (CSC) index, and drug sensitivity assessment. Expression of these hub genes was further validated by RT-qPCR.

**Results:**

We found that most of the PRGs were upregulated in ccRCC tumors with low mutation rates, and 18 PRGs exhibited a significant correlation with ccRCC patient survival. Patients were stratified into three PRG clusters and two gene clusters, which were significantly associated with ccRCC prognosis. We constructed a prognostic risk model based on three genes, dividing ccRCC patients into high- and low-risk groups. The predictive value of this risk model was confirmed by ROC curves. High-risk scores were associated with an increased stromal score, immune score, and tumor mutation burden (TMB), but they were associated with a decrease in the cancer stem cell (CSC) index. RT-qPCR confirmed the expression of *WDR72, ANLN*, and *SLC16A12* in ccRCC tissues and cell lines. Additionally, the PRG risk score model exhibited significant associations with sensitivity to multiple drugs.

**Conclusion:**

This novel PANoptosis-based model addresses the knowledge gap by providing enhanced prognostic accuracy and clinical utility for personalized ccRCC management, potentially guiding targeted and immunotherapeutic strategies.

## Introduction

According to the Global Burden of Disease 2021, renal cell carcinoma (RCC) had an incidence of 434,419 cases in 2022, resulting in 155,702 deaths worldwide (Bray et al., 2024). Clear cell renal cell carcinoma (ccRCC) is the most prevalent subtype, accounting for over 70% of RCC cases (Motzer et al., 2019). Although surgical resection remains the preferred treatment for early-stage ccRCC, approximately 30% of patients are diagnosed at an advanced stage, and 10%–20% develop metastases due to postoperative recurrence, resulting in poor outcomes (Wang B. et al., 2023; Xie et al., 2025). First-line treatments, such as sunitinib, which targets vascular endothelial growth factor receptors (*VEGFRs*) and platelet-derived growth factor receptors (*PDGFRs*), have been developed following the discovery of molecular targets (Faivre et al., 2007). Recent studies have shown that combining pazopanib, a tyrosine kinase inhibitor (*TKI*), with pembrolizumab, an immune checkpoint inhibitor, benefits patients with advanced ccRCC (Rini et al., 2019). Despite these advancements, however, the 5-year overall survival rate for metastatic RCC remains as low as 12% (Barata and Rini, 2017). Therefore, uncovering the genomic characteristics of ccRCC is essential for developing more accurate prognostic signatures and identifying potential therapeutic targets, thereby enhancing personalized treatment and patient outcomes.

Programmed cell death (PCD) is a fundamental physiological process observed in all organisms. Among its well-characterized forms, pyroptosis, apoptosis, and necroptosis have been shown to play crucial roles in cancer, inflammation, infections, and other diseases (Chen et al., 2019). Previous studies have primarily focused on the distinct biological functions and mechanisms underlying these individual forms of PCD. Traditionally, these PCD forms were considered independent, but emerging evidence reveals intricate crosstalk between them (Wang and Kanneganti, 2021). Recently, the concept of PANoptosis was introduced to describe the interconnected mechanisms of pyroptosis, apoptosis, and necroptosis. Unlike traditional forms of cell death, PANoptosis is regulated by a specific multi-protein complex, known as the PANoptosome, which activates these pathways in a coordinated manner (Wang and Kanneganti, 2021). For instance, Caspase-8 is a key protein involved in the PANoptosis crosstalk among pyroptosis, apoptosis, and necroptosis in cancer (Jiang et al., 2021). While several studies have explored PANoptosis in ccRCC, they have primarily focused on non-coding RNAs (e.g., lncRNAs and miRNAs) or immune infiltration patterns using bioinformatics alone, often lacking experimental validation (Wang Y. et al., 2023; Liu et al., 2023). Until now, the role of PANoptosis in ccRCC and its implications for anticancer immunity remain unclear. Therefore, gaining a deeper understanding of the characteristics of PANoptosis is crucial for elucidating the pathophysiological mechanisms of ccRCC and potentially offering novel therapeutic insights.

In this study, we conducted a comprehensive analysis of ccRCC gene expression to explore PANoptosis-related molecular signatures. Subsequently, we analyzed the relationship between the three PANoptosis clusters and features of tumor microenvironment (TME) infiltration, ccRCC prognosis, and HALLMARK gene set variation analysis (GSVA). Patients were then classified into two gene clusters. Patients were classified into two gene clusters, and a novel PANoptosis risk score signature was developed to predict ccRCC prognosis. Additionally, we evaluated the TME, cancer stem cell index, tumor mutational burden (TMB), and drug sensitivities between the two risk groups. Finally, the expression levels of genes in the signature were validated using reverse transcription quantitative polymerase chain reaction (RT-qPCR).

## Materials and methods

### Dataset and preprocessing of ccRCC

**FIGURE 1:**
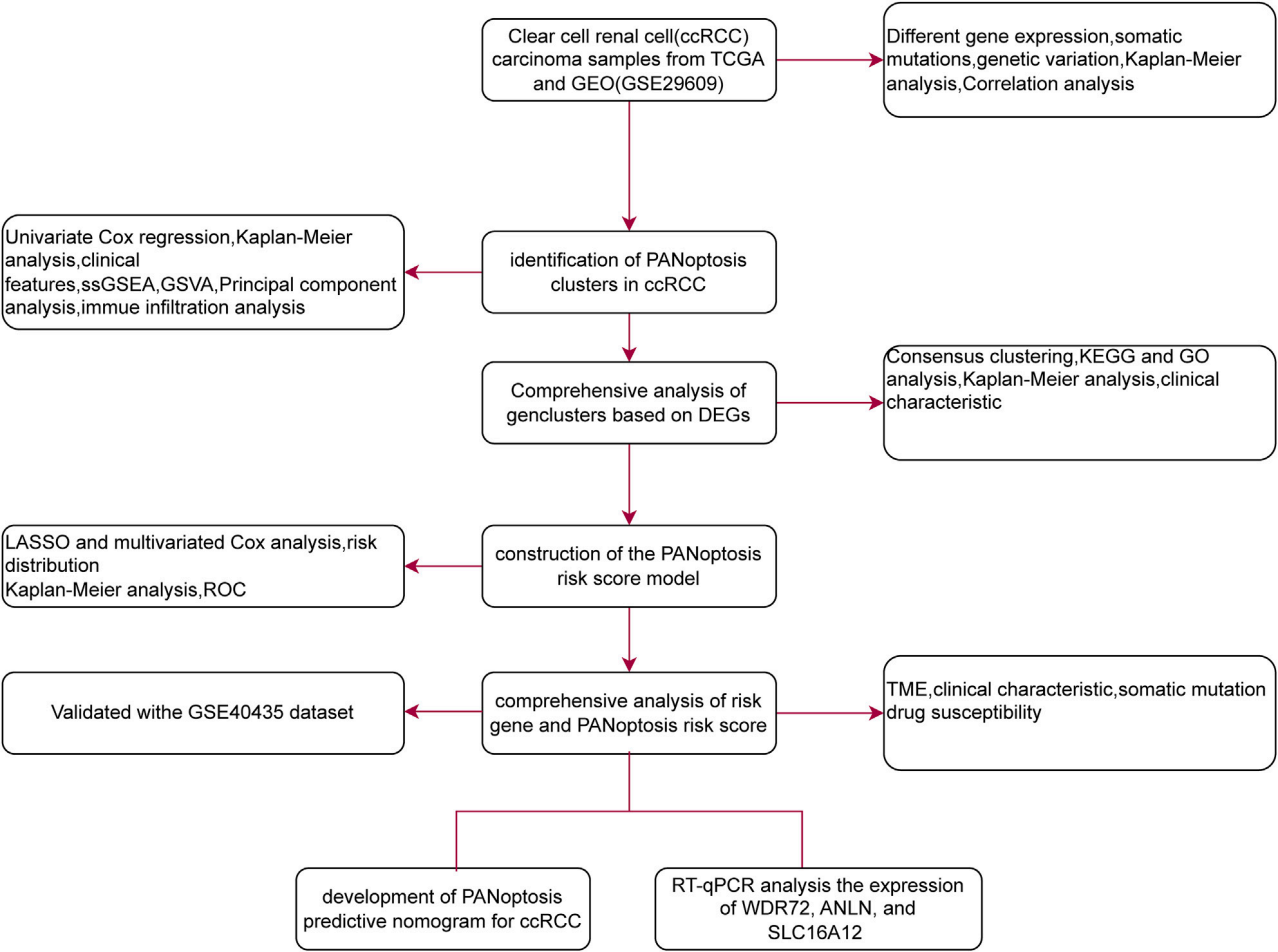
Flowchart of the present study.

RNA-sequencing data, clinical information, and survival data for 524 ccRCC patients and 72 control samples were obtained from The Cancer Genome Atlas (TCGA-KIRC) database. The GEO dataset GSE29609 provided RNA sequencing data for 39 ccRCC patients. We also used the GSE40435 dataset (101 ccRCC and 101 control samples) to validate key findings. To form a unified discovery cohort, TCGA-KIRC and GSE29609 were merged after normalizing expression data to transcripts per million (TPM) values and applying batch effect correction using the ComBat algorithm from the ‘sva’ R package (version 3.42.0). This resulted in a combined cohort of 563 ccRCC samples for primary analyses, including consensus clustering. All processing was conducted in R (version 4.2.2) using the limma (version 3.50.0) and sva (version 3.42.0) packages. Input files were read as tab-delimited matrices with gene symbols in the first column. Common genes across datasets were identified via intersection (resulting in 15,742 genes). For each dataset: (1) Duplicate genes were averaged using avereps from limma. (2) For TCGA-KIRC, only tumor samples were retained by filtering barcodes (fourth digit = 0), duplicate samples averaged if present, and data log2-transformed if quantile checks indicated raw-scale values (qx[5] > 100 or (qx[6] - qx[1]) > 50 & qx[2] > 0), using log2(x + 1). (3) For GSE29609, quantile normalization was applied via normalizeBetweenArrays from limma. Matrices were then merged on intersecting genes, with dataset origin labeled as batch factors. Batch effects were corrected using the ComBat algorithm from sva, with parametric priors (par.prior = TRUE) and dataset as the batch covariate. Quality control metrics included quantile distributions inspected to confirm normalization needs (e.g., median qx values ∼6–10 post-log for TCGA, ∼8–12 for GEO), genes with zero variance or missing in >50% samples filtered via intersection and avereps (∼20% removal), and post-ComBat principal component analysis (PCA) via prcomp confirming batch removal (PC1 now 24.0% variance, PC2 6.6%, together explaining 30.7% variance, with TCGA and GSE29609 samples largely intermixed and clustering by tumor biology; adjusted Rand index pre- vs. post: ∼0.15). This aligns with the workflow depicted in Figure 1, where PCA supports the identification of PANoptosis clusters in ccRCC by validating data integration prior to consensus clustering and subsequent analyses.

### Differential gene expression analysis and identification of prognosis-related PANoptosis

The Wilcoxon rank-sum test was applied to identify differentially expressed PRGs between ccRCC and normal tissues. Prognosis-related PRGs were identified through univariate Cox regression and Kaplan-Meier survival analyses.

### Unsupervised consensus cluster for PANoptosis

Based on 29 PRGs from prior literature (Samir et al., 2020; Wang and Kanneganti, 2021; Wang X. et al., 2022), consensus clustering analysis was conducted using with the “ConsensusClusterPlus” R package to classify ccRCC patients into PANoptosis subtypes. Clustering analysis was performed over 1,000 iterations to ensure robustness, and subtypes were validated using principal component analysis (PCA).

### Gene set variation analysis and enrichment analysis

GSVA was conducted with the “GSVA” R package, using the HALLMARK gene set “h.all.v2022.1.Hs.symbols.gmt” downloaded from the MSigDB database (https://www.gsea-msigdb.org/gsea/index.jsp) to explore biological differences among the subtypes (Liberzon et al., 2015).

In addition, Gene Ontology (GO) and Kyoto Encyclopedia of Genes and Genomes (KEGG) enrichment analyses were performed using the “org.Hs.eg.db” and “enrichplot” R packages to identify relevant biological functions and pathways. An adjusted p value <0.05 indicates statistical significance.

### Establishment and validation of the prognostic signature

Differentially expressed genes (DEGs) were identified between PANoptosis subtypes in pairwise comparisons. DEGs with a log2(fold change) > 0.585 and adjusted p value <0.05 were employed to identify gene signatures. Prognosis-related significant genes were identified through univariate Cox regression analysis. To identify additional PRGs for signature development, we divided the patients into two distinct gene clusters based on the expression of DEGs. To minimize the least absolute shrinkage between signatures, we performed LASSO Cox regression analysis using the univariate significant genes. Subsequently, multivariate Cox regression analysis was conducted to select the best gene and estimate the correlation coefficient. The PRG risk score can be calculated using the following formula: PANoptosis score $=\sum_{i=1}^{n}\exp\left(\text{Xi}\right)\times \text{coef}\left(\text{Xi}\right)$ . In this formula, exp (Xi) represents the expression level of the corresponding genes, and coef (Xi) represents the coefficient of the three genes. Patients were stratified into high- and low-risk groups according to the median risk score, and Kaplan-Meier analysis was performed to evaluate survival differences. Predictive accuracy was validated using time-dependent ROC curves, and a nomogram was developed for 1-, 2-, and 3-year survival prediction, incorporating clinical characteristics and risk groups. Calibration plots were utilized to validate the consistency between the predicted and observed survival rates of patients with ccRCC.

### Relationship between PANoptosis and the TME

Single-sample gene set enrichment analysis (ssGSEA) and the CIBERSORT algorithm were employed to evaluate immune cell infiltration, while the ESTIMATE algorithm quantified stromal and immune scores within the TME which refers to the complex, dynamic environment surrounding a tumor.

### Assessment of mutations and cancer stem cell index

Gene mutation data were analyzed using the “maftools” R package to explore mutation profiles in different groups of ccRCC. Additionally, the relationship between risk scores and the cancer stem cell index was also investigated.

### Prediction of drug sensitivity

Drug sensitivity predictions were made using the “oncoPredict” R package, based on IC50 values from the Genomics of Drug Sensitivity in Cancer v2(GDSC) database for 198 drugs (https://www.cancerrxgene.org/). P-values <0.05 were considered statistically significant.

### RT‒qPCR

A total of 22 pairs of ccRCCs and their adjacent normal samples were collected from Liuzhou Workers’ Hospital, with approval from the Ethics Committee (KY2023412). Total RNA was isolated from the human samples, normal renal cells (Hk-2), and ccRCC cells (Caki-1, Caki-2, A498, 786-o) using TRIzol reagent (Thermo Fisher Scientific, United States) following the manufacturer’s instructions. Reverse transcription was carried out using the PrimeScriptTM RT Reagent Kit (Takara, Japan). Quantitative reverse transcription polymerase chain reaction (qRT‒PCR) was performed on an FX Connect system (Bio-Rad, United States) using SYBR® Green Supermix (Bio-Rad, United States) to measure the expression levels of hub genes. The primer sequences used for qRT‒PCR are provided in Supplementary Table S1. Unpaired t tests were used to assess the differential expression levels between ccRCC and adjacent noncancer tissues. β-Actin was used as the reference gene.

### Statistical analysis

All statistical tests were conducted using R version 4.2.2. For two-group comparisons, the Student’s t-test or Wilcoxon test was applied based on data distribution, while the Kruskal–Wallis test was used for multiple group comparisons. Statistical significance was defined as p < 0.05. In the figures, the asterisks reflect the statistical P value (ns, P ≥ 0.05, *P < 0.05, **P < 0.01, ***P < 0.001, and ****P < 0.0001).

## Results

### Genetic and transcriptional alterations of PANoptosis genes in ccRCC

**FIGURE 2:**
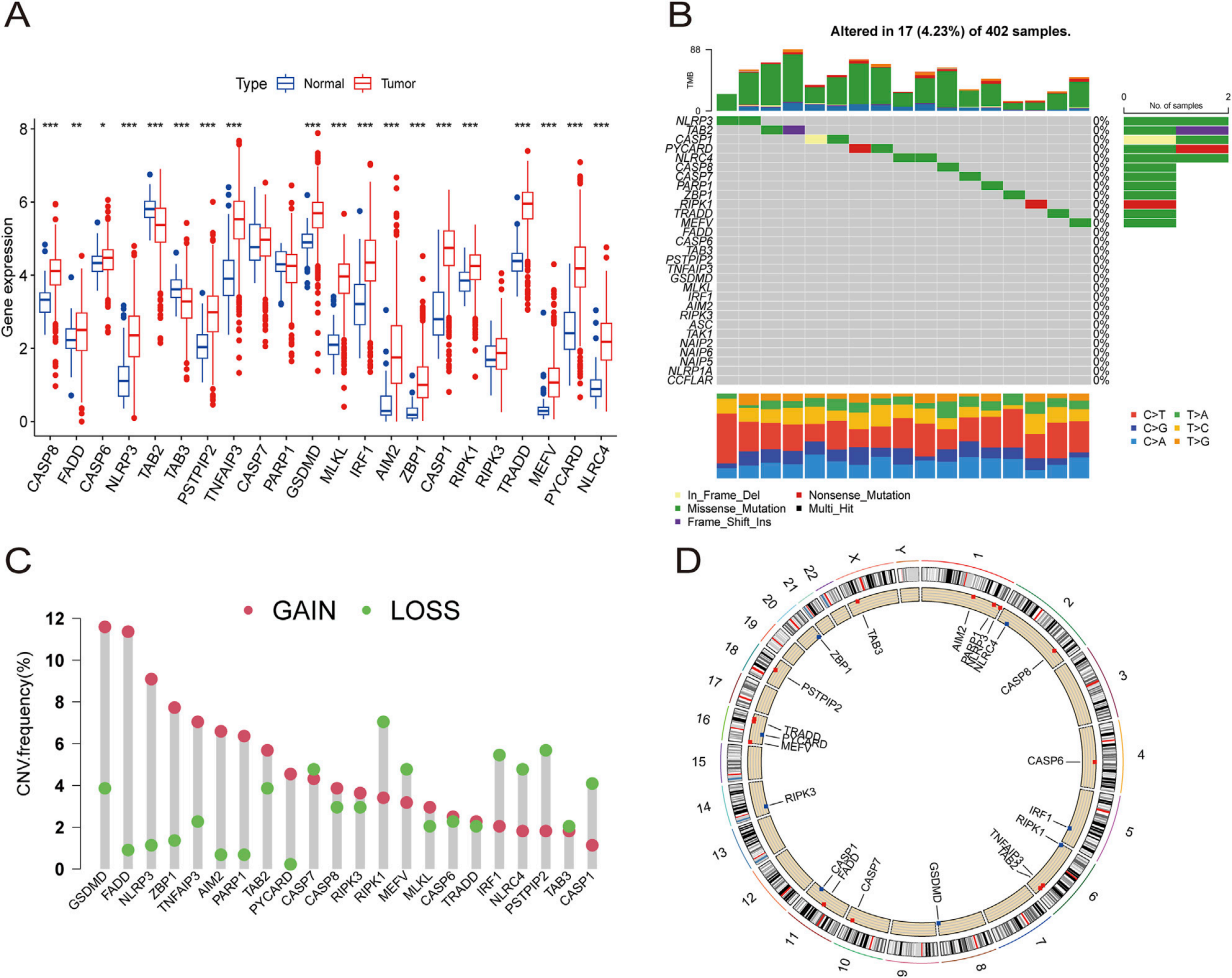
The expression distribution and genetic alteration of PRGs in ccRCC. **(A)** The expression of 29 PRGs in ccRCC samples and normal tissues. **(B)** The mutation frequency and classification of 29 PRGs in ccRCC. **(C)** The CNV variant frequencies of 29 PRGs in ccRCC. **(D)** The locations of PRGs CNV changes on 23 chromosomes.

A total of 29 PRGs were included in this analysis. We compared the differential expression of PRGs in ccRCC tumor samples and healthy samples collected from the TCGA-KIRC database. The results indicated that *CASP8, FADD, CASP6, NLRP3, PSTPIP2, TNFAIP3, GSDMD, MLKL, IRF1, AIM2, ZBP1, CASP1, RIPK1, RIPK3, TRADD, MEFV, PYCARD*, and *NLRC4* were upregulated in ccRCC samples compared to normal samples. However, only TAB2 and TAB3 were downregulated in ccRCC (Figure 2A).

Somatic mutation analysis of the PRGs revealed a low mutation frequency in the ccRCC samples. Only 17 (4.23%) of 402 ccRCC samples exhibited somatic mutations (Figure 2B). Additionally, somatic copy number variation (CNV) analysis was performed on these PRGs, revealing frequent CNV alterations. Among them, *GSDMD, FADD, NLRP3, ZBP1, TNFAIP3, AIM2, PARP1, TAB2, PYCARD, CASP8, RIPK3, MLKL, CASP6*, and *TRADD* showed increased CNV, whereas *CASP7, RIPK1, MEFV, IRF1, NLRC4, PSTPIP2, TAB3*, and *CASP1* showed relatively decreased CNV (Figure 2C). The specific locations of the CNV alterations in PRGs on the chromosomes of PRGs are shown in Figure 2D.

### Identification of PANoptosis molecular subtypes of ccRCC

**FIGURE 3:**
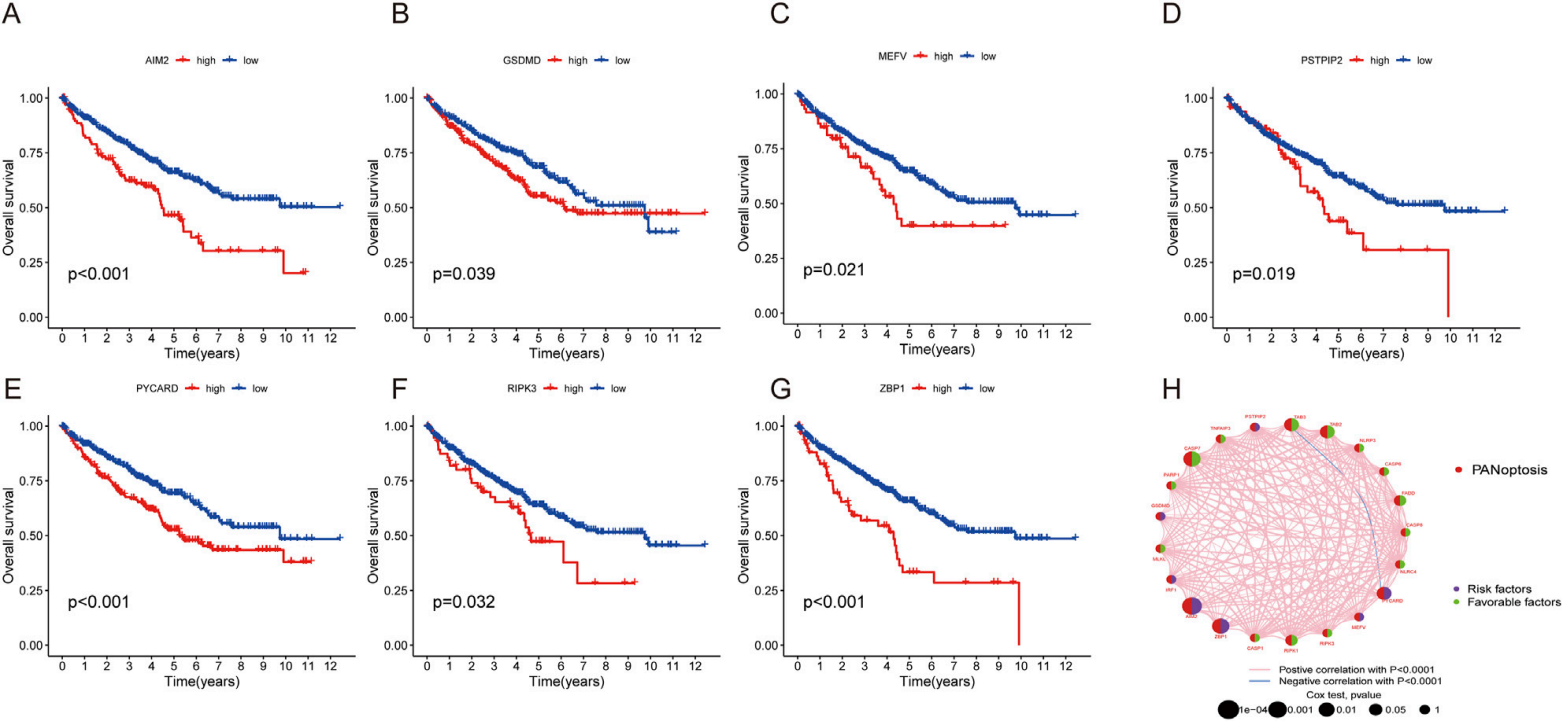
Survival and ROC analysis of the PRGs in ccRCC. **(A–G)** Survival analysis of PRGs (AIM2, GSDMD, MEFV, PSTPIP2, PYCARD, RIPK3, and ZBP1) in ccRCC patients using Kaplan–Meier plots and log-rank tests. **(H)** Network of 29 PRGs in ccRCC.

A total of 524 CCRCC patients from TCGA and 39 patients from the GEO database (GSE29609) were enrolled to explore the expression pattern of PRGs in ccRCC. Kaplan‒Meier curves revealed that 18 PRGs were associated with the overall survival rate (OS) of ccRCC. High expression of *AIM2, GSDMD, MEFV, PSTPIP2, PYCARD, RIPK3, and ZBP1* was associated with poor prognosis, whereas high expression of *CASP1, CASP6, CASP7, FADD, NLRC4, NLRP3, PARP1, RIPK1, TAB2, TAB3*, and *TNFAIP3* was linked to better survival rate than low expression (Figures 3A–G; Supplementary Figure S1). A network was constructed to investigate the intersection between PRGs and their prognostic significance (Figure 3H).

**FIGURE 4:**
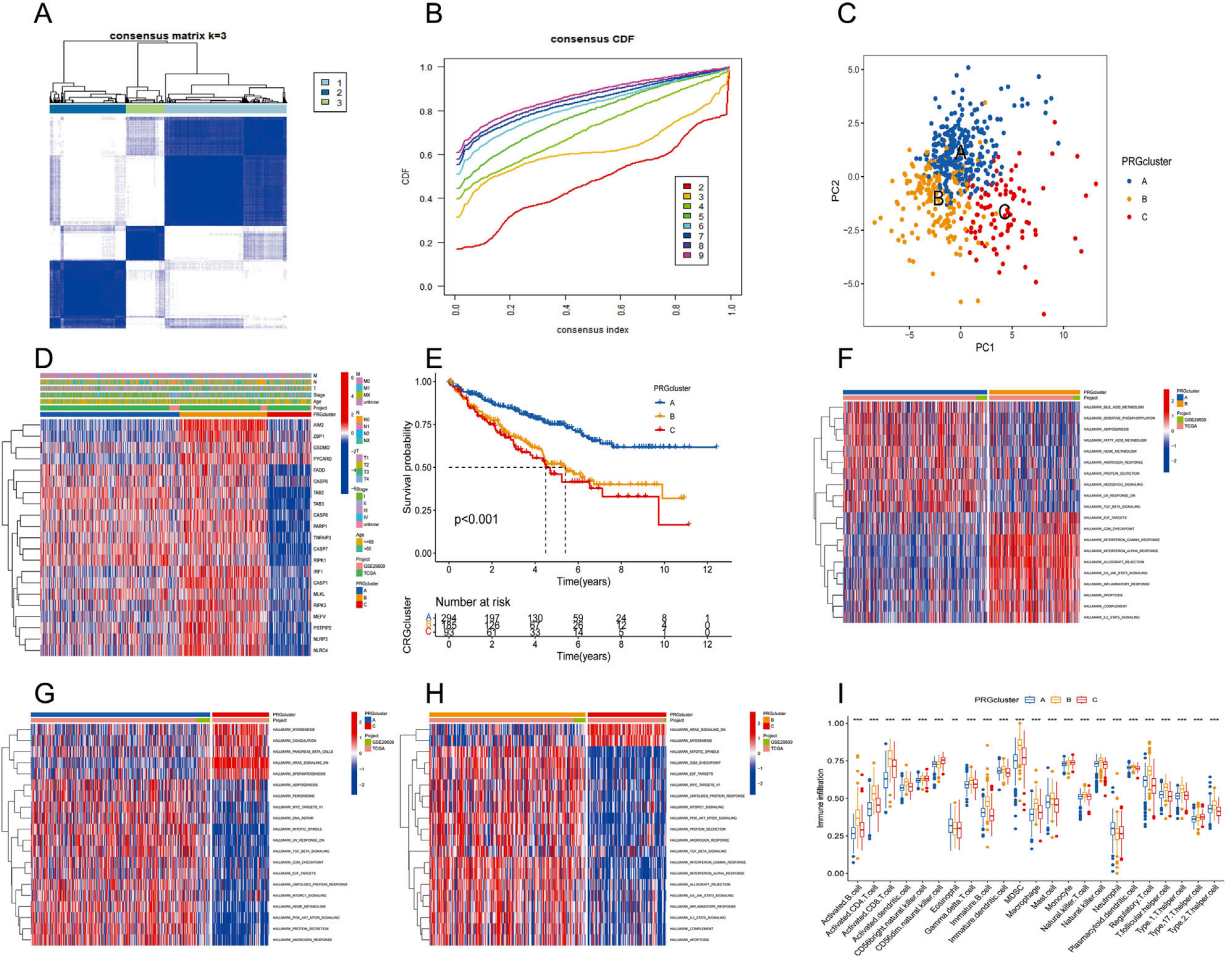
Identification of differential expression of PRGclusters and functional enrichment analysis. **(A,B)** Consensus matrix heatmap defining three clusters (k = 3) and their correlation area. **(C)** Principal component analysis of three PRGclusters. **(D)** A complex heat map demonstrated the expression patterns of 21 PRGs. **(E)** Kaplan–Meier survival curve for the three geneclusters. **(F–H)** Gene set variation analysis showed the differences in biological pathway of three PRGclusters. **(I)** PRGs expression differences between three PRGcluster.

The consensus clustering algorithm was applied to classify 572 ccRCC patients into three molecular subtypes based on PRG expression. Optimal clustering was achieved with K = 3, dividing the patients into three distinct subgroups: PRGcluster A (294 patients), PRGcluster B (185 patients), and PRGcluster C (93 patients) (Figures 4A,B). PCA confirmed the satisfactory separation between these clusters (Figure 4C). We further analyzed the correlation between PRGs expression and TNM stage, clinical stage, and age. The heatmap showed that most PRGs showed relatively high expression in the PRGcluster B group but comparatively lower in the PRGcluster C group (Figure 4D). Survival analyses were also performed between the three PRG clusters. The survival analysis revealed a significant difference in OS between these three PRGclusters, with PRGclusters A showed the best prognosis for ccRCC (Figure 4E).

### TME characteristics in distinct PANoptosis patterns

PRGcluster A was significantly enriched in oxidative phosphorylation, adipogenesis, Hedgehog signaling, and TGF-β signaling, while PRGcluster B was significantly enriched in the *PI3K-AKT-Mtor* pathway, *interferon-β, IL6-JAK-STA3* signaling, complement, and apoptosis. PRGcluster C was significantly enriched in KRAS pathway signaling and myogenesis (Figures 4F–H). Subsequently, ssGSEA was conducted to assess the differences in immune cell infiltration among the three PRGclusters. PRGcluster B was enriched in activated B cells, activated *CD4* T cells, activated *CD8* T cells, activated dendritic cells, *CD56* bright nature killer cells, gamma delta T cells, B immature cells, dendritic immature cells, MDSCs, macrophages, mast cells, natural killer T cells, plasmacytoid dendritic cells, regulatory T cells, T follicular helper cells, type 1 T helper cells, and type 2 T helper cells. PRGcluster A was enriched in eosinophils and neutrophils. While PRGcluster C was enriched in *CD56* dim nature killer cells (Figure 4I).

### Identification of gene signatures based on differentially expressed genes

**FIGURE 5:**
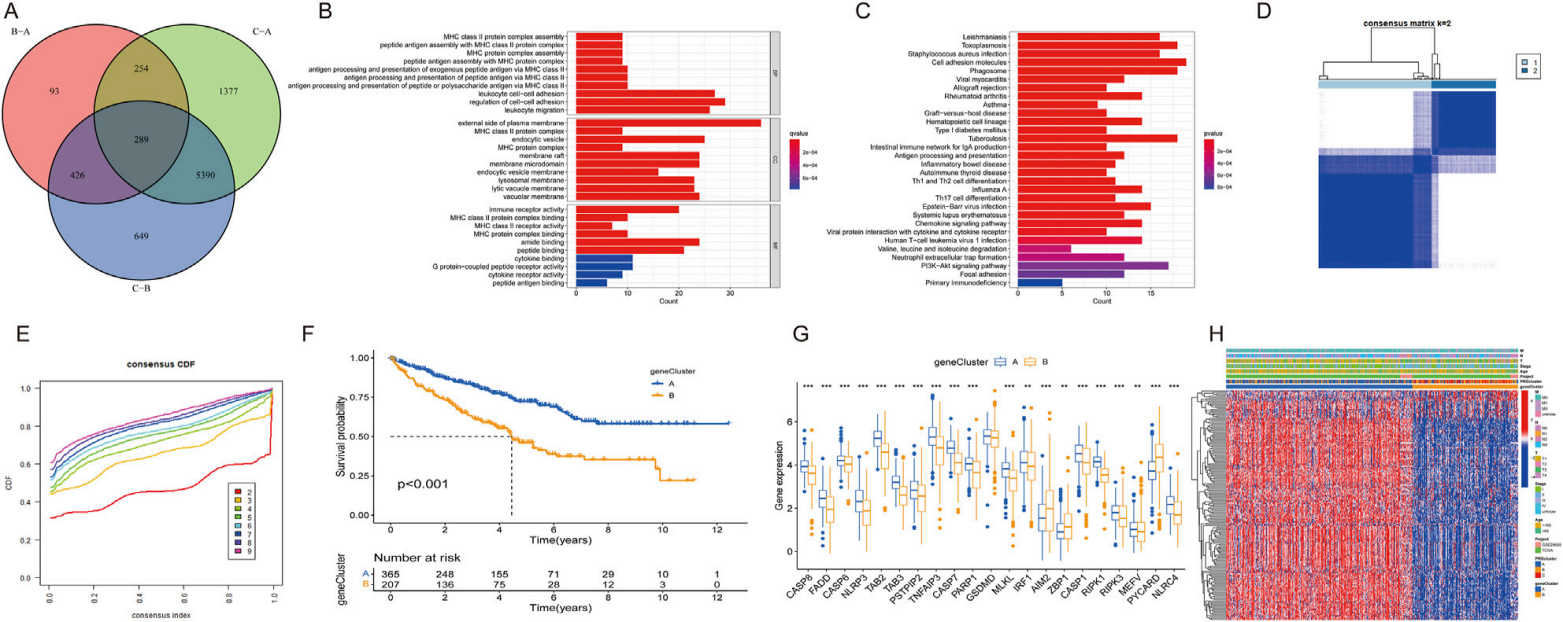
Differential expression of PRG clusters and functional analysis. **(A)** Venn diagram of 289 DEGs. **(B)** GO enrichment analysis of BP, CC, and MF terms. **(C)** KEGG pathway analysis of 289 DEGs. **(D)** Unsupervised clustering of DEGs into two clusters. **(E)** Consensus matrix heatmap of two clusters. **(F)** Kaplan–Meier survival curve for two clusters. **(G)** Expression differences of PRGs between two clusters. **(H)** Heatmap showing expression patterns of 17 DEGs.

DEGs among the three PRGclusters were compared in triplicate to explore the underlying biological functions of these PANoptosis patterns. Across these comparisons, we identified 289 intersecting genes (Figure 5A). Subsequently, we conducted GO and KEGG enrichment analyses on these 289 intersected genes. The GO results indicated that these genes were involved in regulating cell-cell adhesion, leukocyte migration, and leukocyte cell-cell adhesion in the biological process (BP) category. The molecular function (MF) category encompassed amide binding, peptide binding, and immune receptor activity. In the cellular component (CC) category, the genes were significantly associated with the external side of the plasma membrane, endocytic vesicle, and membrane microdomain (Figure 5B). The KEGG results revealed that these genes participated in the PI3K-Akt signaling pathway, focal adhesion, tuberculosis pathways, and cell adhesion molecules (Figure 5C).

Univariate Cox regression analysis identified univariately significant genes. ccRCC patients were classified into two gene subtypes (geneCluster A and geneCluster B) based on the highest correlation coefficient K-value (Figures 5D,E). Survival analysis revealed that ccRCC patients in geneCluster A exhibited better survival outcomes compared to those in geneCluster B (Figure 5F). The boxplot showed that the expression of most PRGs, with the exceptions of *AIM2, ZBP1*, and *PYCARD*, were higher in geneCluster A than in geneCluster B (Figure 5G). A heatmap illustrated the relationships among gene clusters, PRGclusters, and select clinical characteristics (Figure 5H).

### Construction and validation of the PRG risk scoring prognostic signature

**FIGURE 6:**
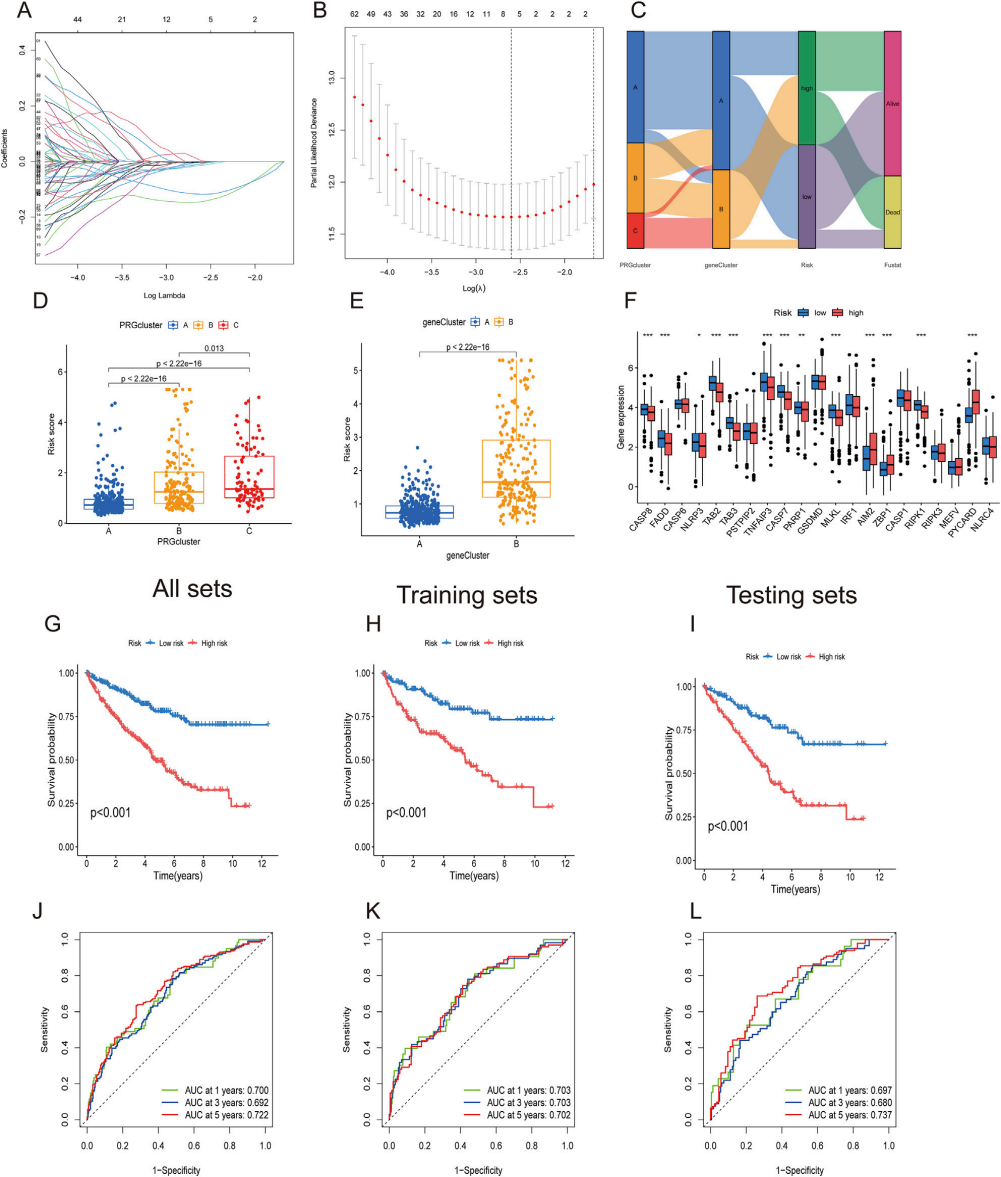
Construction of PANoptosis-related prognostic gene risk model. **(A,B)** LASSO regression identified optimal prognostic genes. **(C)** Alluvial diagram showing changes in PRG clusters, gene clusters, and survival status. **(D,E)** PANoptosis risk score differences between three PRG clusters and two gene clusters. **(F)** Differential PRG expression between high- and low-risk groups. **(G–I)** Kaplan–Meier survival curve for high- and low-risk groups in All, training, and testing sets. **(J–L)** ROC analysis for high- and low-risk groups in All, training, and testing sets.

Considering the complexity and heterogeneity of individual PANoptosis patterns, a risk scoring system incorporating prognosis-related PANoptosis DEGs was constructed to predict PANoptosis patterns in individual ccRCC patients. ccRCC patients were randomly divided into training (n = 286) and testing groups (n = 286) using the “caret” R package. LASSO regression was applied to reduce gene overfitting, resulting in the selection of six significant genes (Figures 6A,B). Multivariate COX regression analysis identified the three top-performing genes (*WDR72, ANLN, and SLC16A12*) The PANoptosis risk scoring formula is as follows: Risk score = Exp (*WDR72*) × (−0.243) + Exp (*ANLN*) × (0.132) + Exp (*SLC16A12*) × (−0.167). All sets consisted of training and testing group files. ccRCC patients were divided into low- and high-risk groups according to their median risk score.

The alluvial diagram depicts changes in the distribution of ccRCC samples (Figure 6C). Figures 6D,E show that PRGcluster C and gene cluster B exhibited higher risk scores. The differential gene analysis of PANoptosis expression between the low- and high-risk groups is presented in Figure 6F. The boxplot results indicated that *AIM2, ZBP1*, and *PYCARD* were the only genes expressed in the high-risk group. The Kaplan‒Meier curve demonstrated that the high-risk group had poorer overall survival probabilities across all sets, including the training and testing sets (Figures 6G–I). ROC curves showed that the AUCs at 1-, 3-, and 5-year survival rates were 0.700, 0.692, and 0.722, respectively, in all sets. In the training set and testing set groups, the AUCs consistently exceeded 0.70 (Figures 6J–L). These findings demonstrated satisfactory predictive accuracy for ccRCC prognosis.

**FIGURE 7:**
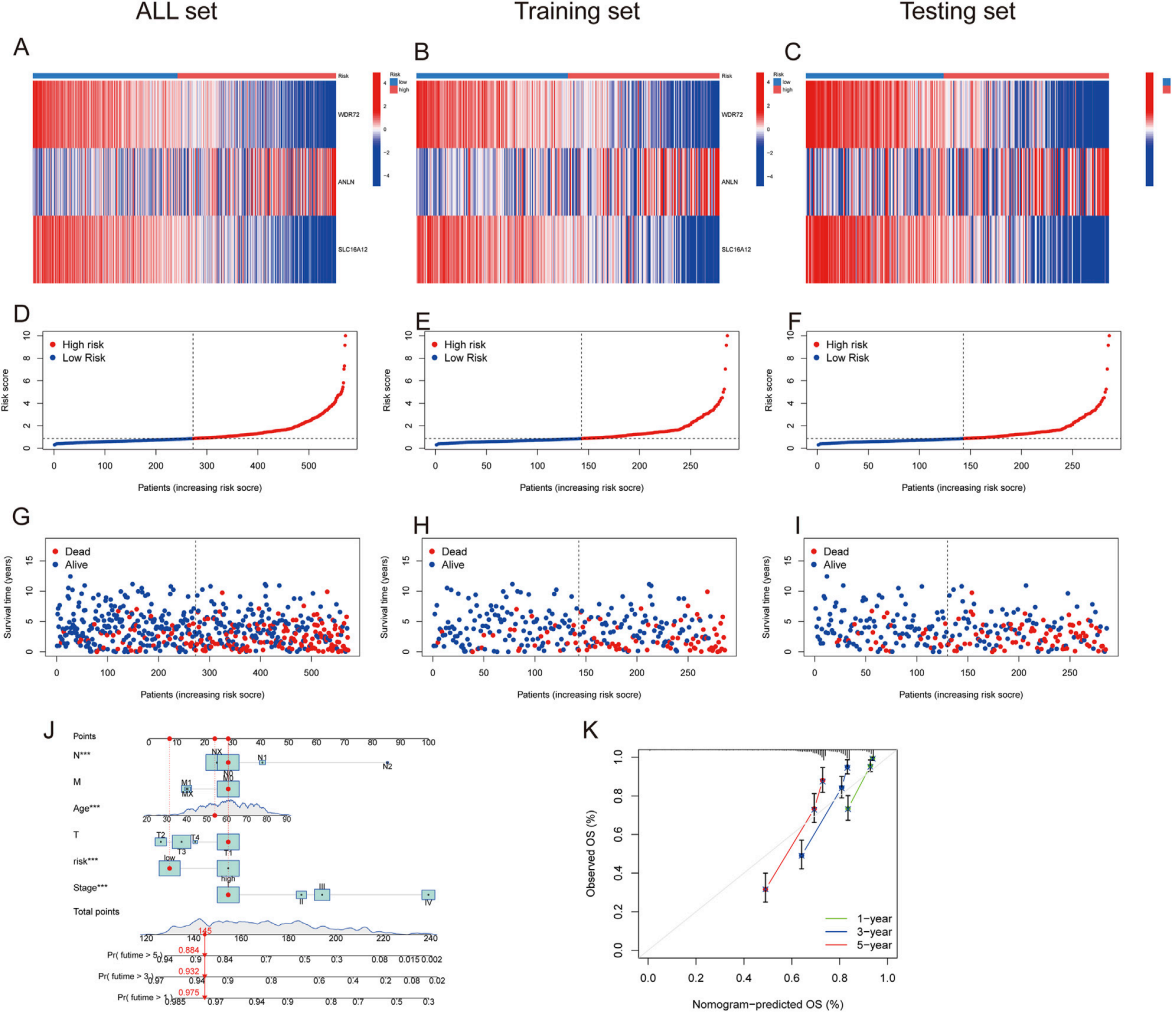
Prognostic value of the risk model in All, training, and testing sets. **(A–C)** Heatmap of three significant genes across different risk scores in All, training, and testing sets. **(D–F)** PRG risk score model in All, training, and testing sets. **(G–I)** Survival status between low- and high-risk groups in All, training, and testing sets. **(J)** Nomogram of the risk score and clinical parameters for all sets. **(K)** Calibration curves showing nomogram accuracy at 1-, 2-, and 3-years in TCGA and GEO datasets.

Figures 7A–C depict the differences in *WDR72, ANLN*, and *SLC16A12* expression levels across all datasets, including the training and testing sets. The heatmap results indicated that *WDR72* and *SLC16A12* were highly expressed in the low-risk group across all three datasets. Across all datasets, including the training and testing sets, the risk score was negatively associated with survival times, indicating that ccRCC patients with lower scores experienced better survival outcomes (Figures 7D–J). A nomogram incorporating the PRG risk score, age, and clinical characteristics was constructed to predict 1-, 3-, and 5-year survival outcomes (Figure 7J). The calibration curve indicated a strong correlation between predicted and observed OS (Figure 7K).

### Relationship between the tumor microenvironment and high- and low-risk groups

**FIGURE 8:**
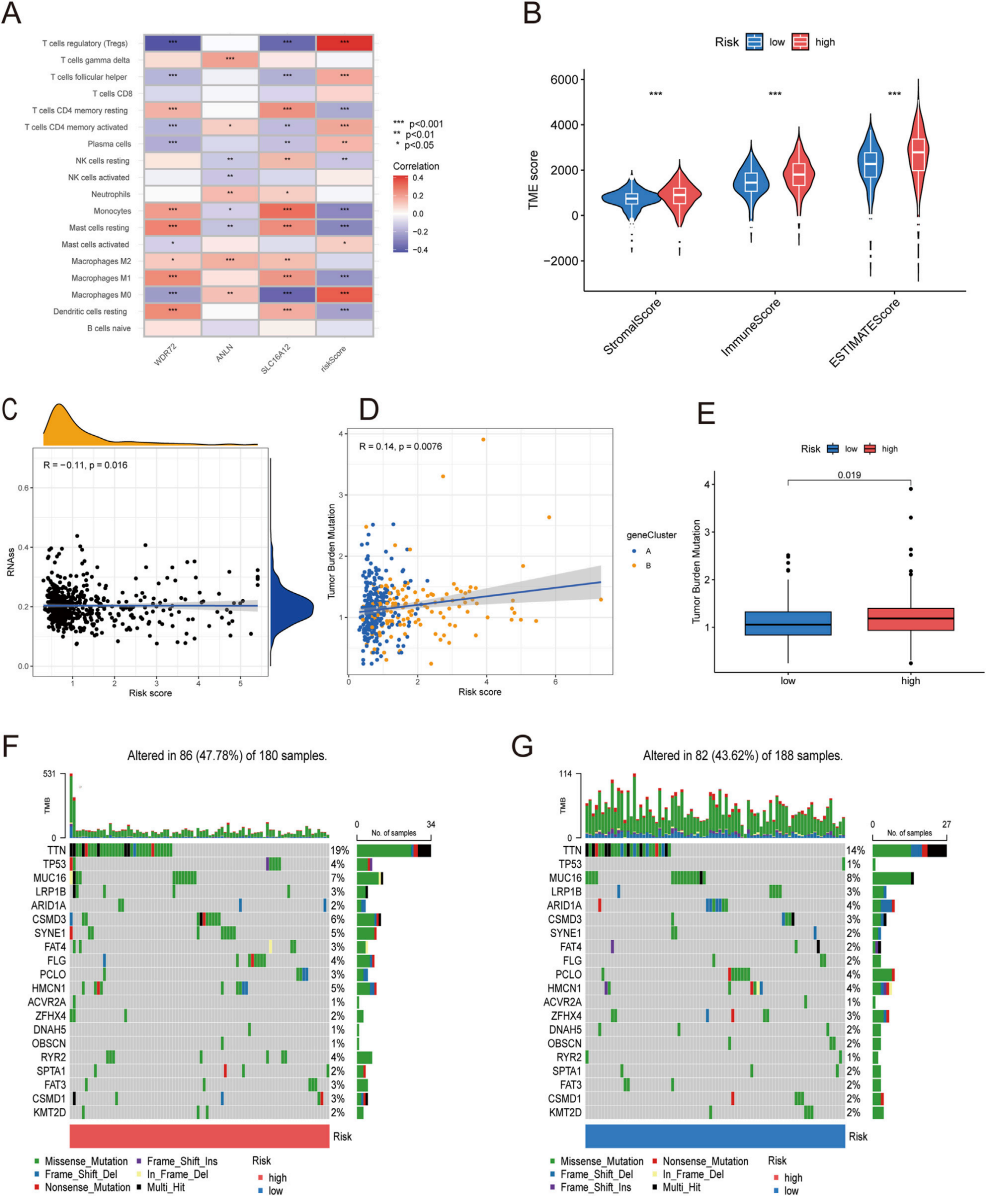
Correlation between PANoptosis risk score and immune cells, CSC, TMB, and TME. **(A)** Correlation between immune cell abundance and risk genes. **(B)** Differential analysis of stromal, immune, and estimate scores. **(C)** Correlation between CSC index and PANoptosis risk score. **(D)** Correlation between TMB and PANoptosis risk score. **(E)** Differential TMB analysis in high- and low-risk groups. **(F,G)** Somatic mutation signatures in high- and low-risk groups.

The results of correlation analyses between the 22 immune cells and the risk score showed that the risk score was positively correlated with M0 macrophages, mast cells, plasma cells, CD4 memory-activated T cells, follicular helper T cells, and Tregs but negatively correlated with resting dendritic cells, M1 macrophages, resting mast cells, monocytes, resting NK cells, and CD4 memory resting T cells (Supplementary Figure S2). Subsequently, correlations between the three risk genes and immune cell populations were examined, revealing associations between certain genes and specific immune cells (Figure 8A). Results indicated that *ANLN* expression positively correlated with M2 macrophage infiltration, a hallmark of immune evasion and immunotherapy resistance. *WDR72* may facilitate TME remodeling by negatively correlating with activated memory CD4^+^ T cells and positively with resting CD4 memory T cells, influencing immune checkpoint blockade efficacy and possibly leading to therapy resistance via altered T cell dynamics. *SLC16A12*, involved in metabolite transport, shows positive correlation with M2 macrophages and negative correlation with M0 macrophages, suggesting a role in macrophage phenotype modulation that could enhance TME remodeling toward a less aggressive state, potentially improving prognosis but also affecting sensitivity to certain therapies. Furthermore, ccRCC patients in the high-risk group exhibited significantly higher stromal, immune, and ESTIMATE score than those in the low-risk group (Figure 8B).

### The associations between the risk score and TMB, CSC, and drug susceptibility

Correlation analysis between the risk score and cancer stem cells (CSCs) revealed a significant negative association (Figure 8C). Tumors with low TMB are typically linked to poor prognosis and limited responsiveness to immunotherapy. Our findings indicated that the low-risk group exhibited a lower TMB than the high-risk group, and the risk score was positively correlated with TMB (R = 0.14, p = 0.0076) (Figures 8D,E). Waterfall plots were generated to compare mutation frequencies between the high- and low-risk groups. In the high-risk group, *TTN, MUC16, CSMD3, SYNE1, and HMCN1* were the five most frequently mutated genes. In the low-risk group, *TTN, MUC16, ARID1A, PCLO*, and *HMCN1* represented the top five most frequently mutated genes. Overall, the high-risk group exhibited a higher mutation frequency than the low-risk group (Figures 8F,G).

**FIGURE 9:**
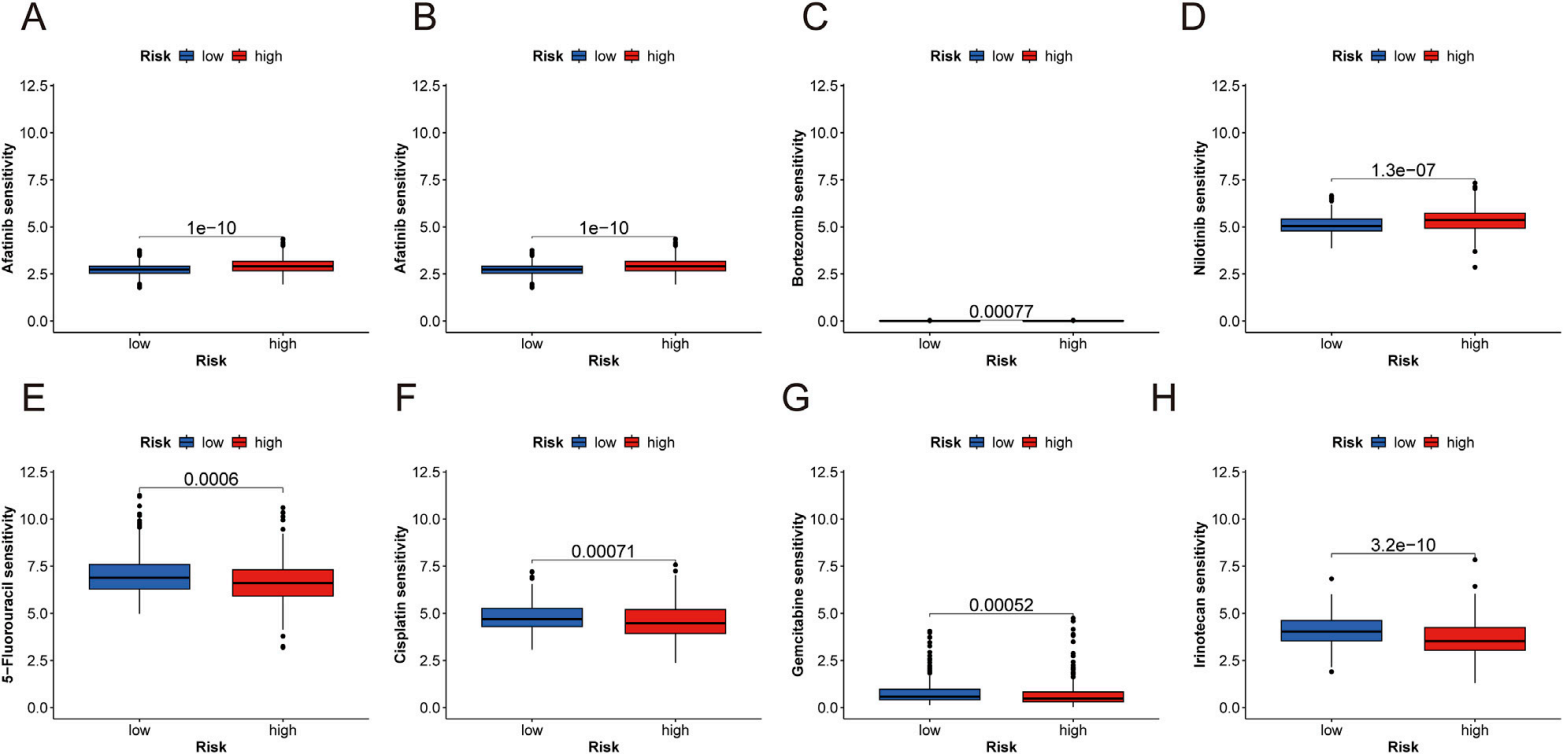
Anticancer sensitivity analysis and RT-qPCR analysis of three risk genes. **(A–H)** Examples prediction of antitumor drug sensitivity for high- and low-risk groups (afatinib, axitinib, bortezomib, and nilotinib, 5-fluorouracil, cisplatin, irinotecan, and gemcitabine).

Drug sensitivity was compared between the low- and high-risk groups to evaluate the signature’s predictive efficacy for ccRCC treatments (Supplementary Table S3). Our study found that low-risk patients exhibited greater sensitivity to afatinib, axitinib, bortezomib, and nilotinib. Conversely, high-risk patients displayed significantly lower IC50 values for 5-fluorouracil, cisplatin, irinotecan, and gemcitabine. These findings indicate that the PANoptosis signature could be useful in predicting the drug susceptibility of ccRCC patients (Figures 9A–H).

### Validation of the expression levels of *WDR72, ANLN*, and *SLC16A12* via RT‒qPCR

**FIGURE 10:**
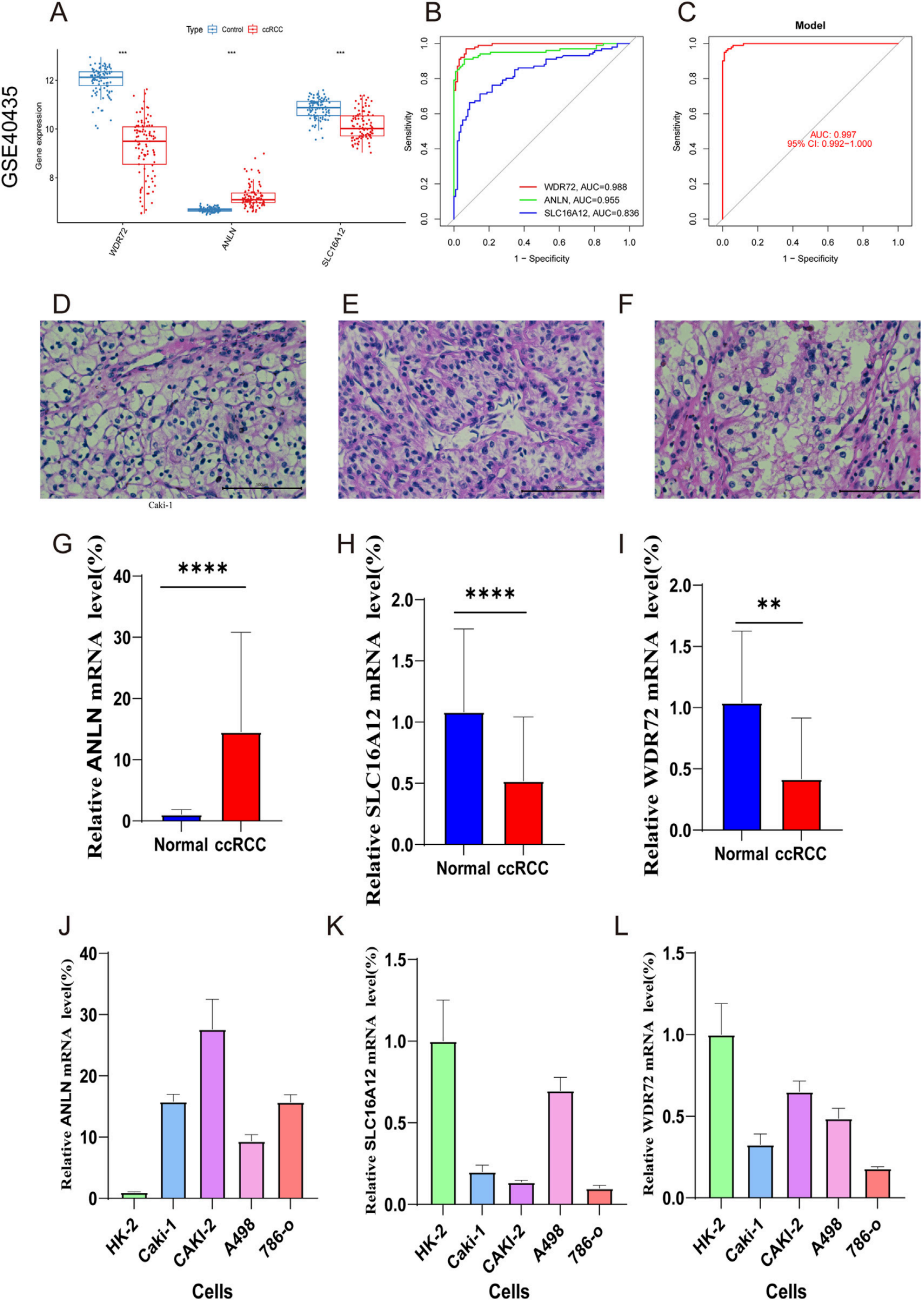
External validation with GSE40435 dataset and RT-qPCR validation of hub genes. **(A)** Expression of hub genes in ccRCC and control samples. **(B)** ROC analysis of the marker genes. **(C)** ROC analysis of the three-gene model with 3-fold cross-validation. **(D–F)** HE staining confirming ccRCC diagnosis. **(G–I)** RT-qPCR of ANLN, SLC16A12, and WDR72 in ccRCC and normal samples. **(J–L)** qRT-PCR validation of marker genes in ccRCC and normal renal cells.

Additionally, the expression levels of the three marker genes and their ROC curve performance were verified using the GSE40435 dataset. The findings showed increased expression levels for *ANLN*, while *WDR72* and *SLC16A12* exhibited decreased expression levels (Figure 10A). As shown in Figure 10B, all three hub genes achieved ROC curve AUC values above 0.80 in the GSE40435 dataset (*WDR72*, AUC = 0.998; *ANLN*, AUC = 0.955; *SLC16A12*, AUC = 0.836). Additionally, the combined AUC for three hub genes was higher than that of any single gene, indicating improved predictive accuracy (AUC = 0.997) (Figure 10C). These findings suggest that the three marker genes may serve as potential diagnostic biomarkers for ccRCC.

Figures 10D–F validated the ccRCC diagnosis. RT‒qPCR was then conducted to compare mRNA expression levels of *WDR72, ANLN*, and *SLC16A12* in adjacent normal and ccRCC tumor tissues. The results revealed ANLN upregulation and *SLC16A12* and *WDR72* downregulation in ccRCC tumor tissues compared to adjacent normal tissues (Figures 10G–I). We also examined the expression of the three marker genes in normal renal cell (Hk-2) and ccRCC cells (Caki-1, Caki-2, A498, 786-o). The result indicated that compared to HK-2 cells, *ANLN* expression was upregulated, whereas *SLC16A12* and *WDR72* were downregulated in ccRCC cell lines (Figures 10J–L).

## Discussion

PANoptosis, integrating pyroptosis, apoptosis, and necroptosis, is a novel cell death mechanism with emerging relevance in cancer (Wang and Kanneganti, 2021; Pandian and Kanneganti, 2022). Although some studies have constructed prognostic models and molecular signatures based on PANoptosis-related lncRNA and miRNA, with a focus on immune infiltration and prognosis prediction. However, these models relied solely on bioinformatics without experimental validation, limiting their clinical applicability. Our study comprehensively investigated PANoptosis-related genes (PRGs) in ccRCC, addressing a critical knowledge gap.

In this study, we analyzed 29 PRGs and examined their CNVs, somatic mutations, differential gene expression levels, biological pathways, and PPI networks. Our findings suggest that CNV may alter the expression of many PANoptosis-related genes. Most PANoptosis genes were upregulated in ccRCC, while *TAB2* and *TAB3* were found to be downregulated. The downregulation of *TAB2* and *TAB3* in ccRCC was further validated using the GEPIA database. Conversely, *TAB3* was reported to be upregulated in hepatocellular carcinoma and colorectal cancer (Luo et al., 2017; Wen et al., 2022). Furthermore, 18 PRGs were significantly associated with the overall survival of ccRCC patients. These findings collectively suggest that PANoptosis is a critical factor in the pathophysiology and prognosis of ccRCC. Analysis using ssGSEA and GSVA revealed that PRGcluster B displayed substantial immune cell infiltration and significant enrichment in immune-related pathways. PRGcluster B was also enriched in cancer-related signaling pathways, such as the *PI3K/AKT/mTOR* pathway and complement pathway. Previous research has shown that *PI3K* and *mTOR* are involved in regulating immune cell activation, including mast cells, myeloid immune cells, and neutrophils (Weichhart and Säemann, 2008). Thus, detailed studies of PRGs could aid in developing a more accurate prognostic model to predict ccRCC survival outcomes. Furthermore, understanding the relationship between PRGs, particularly among different PRGcluster groups, and the TME could help clinicians assess immunotherapy effectiveness.

In light of the heterogeneity of PRGs, we developed a risk score model. Compared to previous models of immune-related PANoptosis lncRNAs in ccRCC, our model showed a higher AUC value, indicating more accurate diagnostic capability for ccRCC (Liu et al., 2023). However, with AUC values ranging from 0.680 to 0.737 across different time points still have some reasons and limitations. Several factors may explain this limitation. First, ccRCC is a highly heterogeneous malignancy, and single-cohort transcriptomic signatures may not fully capture its biological and clinical diversity. Second, our study relied on publicly available datasets (TCGA and GEO), which may introduce selection bias and restrict model generalizability to broader populations. Third, although our model integrates immune, TMB, and CSC analyses, only three hub genes (WDR72, ANLN, SLC16A12) were retained after LASSO and multivariate Cox regression, which improves interpretability but may limit predictive power compared to more complex multi-gene models. Additionally, we confirmed the expression of the three hub genes via qPCR analysis of human ccRCC tissue, unlike the PANoptosis-related miRNA model by Yanmei Wang et al., which lacked experimental validation (Wang Y. et al., 2023). Nomograms, widely used in oncology to predict survival outcomes, incorporated risk scores and various clinical characteristics to provide more precise survival estimates. Thus, individual ccRCC patients can use our nomogram to predict their 1-, 3-, and 5-year OS based on TNM stage, age, and PANoptosis risk score. High-risk patients, identified by poor prognosis (high-risk scores), can be prioritized for aggressive therapies such as targeted therapies (e.g., *VEGF* and *mTOR* inhibitors) or immune checkpoint inhibitors (e.g., PD-1/PD-L1 inhibitors). For instance, high-risk patients with elevated immune scores and TMB may be more suitable for immune checkpoint inhibitors like pembrolizumab combined with axitinib, potentially overcoming therapy resistance linked to hub genes such as *ANLN*’s role in immune evasion. Conversely, low-risk patients, characterized by lower TMB and CSC index, might respond better to TKIs such as sunitinib or pazopanib as monotherapy, based on the model’s drug sensitivity predictions and TME insights. This stratified approach enhances clinical decision-making by tailoring treatments to risk profiles, optimizing efficacy, reducing adverse effects, and improving resource allocation. For these patients, timely intervention is crucial, and the model could aid clinicians in making earlier treatment decisions. Conversely, patients with a low-risk score may benefit from more conservative treatment, minimizing unnecessary exposure to toxic therapies and improving quality of life. This approach can also help optimize healthcare resources by prioritizing patients with the greatest need.

*WDR72* has been recognized as a prognostic biomarker in non-small cell lung cancer, where it modulates the *AKT/HIF-1α* signaling pathway, contributing to enhanced lung cancer stemness (Ouyang et al., 2022; Shi et al., 2023). *WDR72*, which encodes proteins that facilitate the formation of multiprotein complexes, has also been identified as a potential therapeutic target and prognostic marker in renal cell carcinoma. Elevated *WDR72* expression has been shown to inhibit cell proliferation and invasion in renal cell carcinoma, supporting its role as a tumor suppressor (Zou et al., 2020). Immunohistochemistry studies have reported lower *WDR72* expression in ccRCC tissue compared to normal renal tissue (Wu et al., 2021), a result we confirmed via RT-qPCR. ANLN, an actin-binding protein, is essential for cell division, growth, migration, and cytokinesis (Shi et al., 2022; Zhang et al., 2022). In gastrointestinal cancers—including hepatocellular carcinoma, gastric cancer, and colorectal cancer-*ANLN* mRNA levels are elevated compared to normal cell lines (Shi et al., 2022). Prior studies have linked elevated *ANLN* expression in ccRCC to poor prognosis (Chen et al., 2022). Our findings confirmed that *ANLN* expression levels were higher in ccRCC tissues than in normal kidney tissues. *SLC16A12*, predominantly expressed in renal tissues, mediates creatine transport (Mei et al., 2019). Previous studies have found that increased *SLC16A12* expression correlates with improved survival rates in ccRCC (Mei et al., 2019; Hou and Liu, 2023), highlighting its potential as a prognostic biomarker and therapeutic target (Hou and Liu, 2023). Thus, these three hub genes represent promising biomarkers for ccRCC prognosis and potential therapeutic targets.

Immunoreactivity is a critical factor in tumorigenesis and offers a promising therapeutic target in oncology (Wang D.-R. et al., 2022). This study investigated associations between the risk score, the three signature genes, and immune cells. Results showed that five immune cell types positively correlated with the risk score, while six exhibited negative correlations. Increasing evidence indicates that stromal cells are critical in tumor growth, progression, differentiation, and cancer therapy (Hanahan and Weinberg, 2011; Straussman et al., 2012). A high stromal score is recognized as a risk factor for poor prognosis (Panayiotou et al., 2015). Infiltrating immune cells demonstrate context-dependent roles associated with tumor invasion and metastasis (Fridman et al., 2012). Prior research has shown that a high immune score correlates with improved chemotherapy and immunotherapy outcomes (Dai et al., 2020). The low-risk group displayed lower immune and stromal scores, suggesting better prognosis but poorer response to antitumor therapies. *WDR72* was negatively correlated with activated memory CD4^+^ T cells and positively associated with resting CD4 memory T cells, consistent with findings in gastric cancer (Zheng et al., 2024). Previous studies showed that proliferative CD4^+^ T cells can drive immune checkpoint blockade (ICB) therapy resistance in ccRCC via TME modulation and tumor cytolysis (Zheng et al., 2022). This suggests that *WDR72* may influence CD4^+^ T cell activity, potentially affecting ICB therapy efficacy. We observed a significant correlation between M2 macrophages and *ANLN* expression. Prior research has also shown that *ANLN* is positively associated with CD163, a marker of M2 macrophages, suggesting that ANLN may influence M2 macrophage polarization, thereby affecting ccRCC immunotherapy responses (Gao et al., 2024). *SLC16A12* was positively correlated with M2 macrophages and negatively correlated with M0 macrophages. Additionally, *SLC16A12* was negatively correlated with PANoptosis. Previous research indicated that M0 macrophages are early-stage M2 macrophages and are associated with poor prognosis in ccRCC (Farha et al., 2023). This suggests that *SLC16A12* may improve ccRCC prognosis by reducing M0 macrophages and increasing M2 macrophages.

CSCs drive tumor progression and growth, contributing to recurrence, metastasis, and drug resistance (Yu et al., 2012; Walcher et al., 2020). Intriguingly, we observed a negative correlation between the risk score and CSC index, a finding that appears counterintuitive given that high stemness is often linked to poor outcomes. We propose this reflects two distinct biological phenotypes. The high-risk group, characterized by a higher TMB and a more inflamed tumor microenvironment, may represent a highly proliferative tumor subtype. This rapid cell division and active immune milieu could be less favorable for maintaining a stable, quiescent CSC population (Celià-Terrassa and Jolly, 2020). Conversely, the low-risk group, while demonstrating better overall survival, had a higher CSC index. This could signify a more indolent, stem-cell-driven tumor that relies on its CSC pool for long-term persistence and is associated with the potential for therapy resistance and future recurrence (Makena et al., 2020).

Lower IC50 values for both chemotherapeutic and immunotherapeutic drugs were observed in the low-risk group, with only 38 drugs exhibiting higher sensitivity in the high-risk group. The discrepancy between drug prediction and TME-related analysis could be due to the exclusion of certain high-risk-sensitive drugs from our dataset. The PANoptosis risk score model, integrating *WDR72, ANLN,* and *SLC16A12* expression, shows promise for personalizing ccRCC treatment. Including drug sensitivity analysis in the PANoptosis risk score model may help identify patients whose tumors are more likely to respond to specific chemotherapeutics. This approach could improve treatment outcomes while minimizing unnecessary therapies and their associated side effects. In conclusion, these findings may aid clinicians in selecting appropriate drugs for ccRCC treatment and offer new insights into PANoptosis’s role in ccRCC. RT-qPCR validated differential expression of *WDR72, ANLN,* and *SLC16A12* in normal renal versus ccRCC tumor tissues, supporting their potential as diagnostic and therapeutic biomarkers.

In the broader context of PANoptosis research in ccRCC, our findings build upon and extend prior models. Unlike the 21-gene signature in Jiang et al. (2024), which highlight ted VHL/BAP1 alterations but limited drug predictions to sunitinib/paclitaxel, or the 5-gene model in Yue et al. (2024) with a nomogram lacking CSC integration, we incorporated 29 PRGs for multi-tiered clustering and uniquely linked high-risk scores to CSC suppression alongside TME/TMB elevations. The miRNA focus of Wang Y. et al. (2023) and lncRNA/single-cell emphasis in Liu et al. (2023) advanced immune checkpoint insights but overlooked broad chemotherapy sensitivities, while (Xu et al., 2024) PANII across RCC subtypes did not delve into ccRCC-specific DEGs or wet-lab validation. By bridging these gaps with hierarchical stratification, CSC-TME interplay, eight-drug predictions, and RT-qPCR in multiple cell lines, our work offers enhanced prognostic precision and therapeutic actionable insights, positioning it as a complementary advance for personalized ccRCC management.

This study has several limitations that need to be addressed in future research. The reliance on publicly available datasets, such as TCGA and GEO, introduces potential selection bias, which may affect the accuracy and generalizability of our findings. Specifically, while the external GEO dataset (GSE40435) was valuable for validating the expression and diagnostic potential of the three individual hub genes, it lacked the complete clinical survival data required for a full external validation of our prognostic risk model. This represents a significant gap, and future studies should prioritize testing the model on independent cohorts with comprehensive follow-up information to confirm its prognostic robustness. Second, our experimental validation was restricted to RT-qPCR analysis, future work will use immunohistochemistry or Western blot to validate hub gene protein expression in ccRCC tissues; moreover, it also highlighting the need for more extensive *in vitro* and *in vivo* studies to further explore the functional roles of PANoptosis-related genes in ccRCC and their interactions within the TME. Further investigations should validate the prognostic signature and examine immunotherapy outcome differences between high- and low-risk patients. Future research should incorporate patient-derived organoids and animal models to improve understanding of these mechanisms. Integrating multi-omics data and advanced machine learning approaches could refine the risk model, improving its clinical predictive accuracy and enabling more personalized treatment strategies, especially in assessing immunotherapy sensitivity and resistance in ccRCC subtypes.

## Conclusion

In conclusion, we established a novel prognostic PRG signature and molecular clustering model that improves ccRCC prognosis prediction. The PANoptosis risk model not only evaluates individual ccRCC patient prognosis but also offers insights into TME cell infiltration patterns. Our findings emphasize the clinical importance of PANoptosis across ccRCC molecular subtypes, providing a deeper understanding of its role in disease progression. Additionally, our analysis identified *WDR72, ANLN,* and *SLC16A12* as promising therapeutic targets. Targeting these genes or their upstream regulators could increase tumor cell sensitivity to treatment. This foundation supports personalized treatment strategies for various ccRCC subtypes based on the risk scores of these three hub genes.

## Data Availability

The original contributions presented in the study are publicly available. This data can be found here: the TCGA-KIRC (https://portal.gdc.cancer.gov/) and in GEO (https://www.ncbi.nlm.nih.gov/geo/) with the accession number GSE29609.
