# Supplementary material for: PANoptosis-related gene clusters and prognostic risk model in clear cell renal cell carcinoma
Source: Front Genet. 2025 Nov 18;16:1605078. doi: 10.3389/fgene.2025.1605078 (PMC12668651; doi:10.3389/fgene.2025.1605078)

**Identification of PANoptosis-related molecular pattern, construction of a prognosis signature predicts prognosis and tumor microenvironment landscape in clear cell renal cell carcinoma**

| Table S1. The primers of β-actin, ANLN, WDR72, and SLC16A12 | | |
| --- | --- | --- |
| β-actin | Forward | CACCATTGGCAATGAGCGGTTC |
|  | Reverse | AGGTCTTTGCGGATGTCCACGT |
| ANLN | Forward | CAGACAGTTCCATCCAAGGGAG |
|  | Reverse | CTTGACAACGCTCTCCAAAGCG |
| WDR72 | Forward | CTGTGAGGATGATAAAATGGCACC |
|  | Reverse | CGTGCTCTTTCTCCTGTCTCATG |
| SLC16A12 | Forward | GACCACCTCTTTGTCATCAGCG |
|  | Reverse | ATCCACAGAGGAGGAATGCTGC |

| Table S2. The prognostic values of 21 DEGs in ccRCC patients with a univariate Cox regression model. | | | | |
| --- | --- | --- | --- | --- |
| Genes | HR | HR.95L | HR.95H | pvalue |
| CASP8 | 0.958383 | 0.748988 | 1.226318 | 0.735396 |
| FADD | 0.808242 | 0.66837 | 0.977385 | 0.028099 |
| CASP6 | 0.815802 | 0.599352 | 1.11042 | 0.195611 |
| NLRP3 | 0.860092 | 0.717836 | 1.03054 | 0.102291 |
| TAB2 | 0.738649 | 0.615963 | 0.885771 | 0.00108 |
| TAB3 | 0.686025 | 0.54321 | 0.866388 | 0.001555 |
| PSTPIP2 | 1.099593 | 0.908254 | 1.33124 | 0.330373 |
| TNFAIP3 | 0.873537 | 0.749884 | 1.017581 | 0.082534 |
| CASP7 | 0.698861 | 0.57438 | 0.850321 | 0.000344 |
| PARP1 | 0.842773 | 0.679656 | 1.045038 | 0.119096 |
| GSDMD | 1.134906 | 0.884619 | 1.456008 | 0.31948 |
| MLKL | 0.957272 | 0.786846 | 1.164611 | 0.662439 |
| IRF1 | 1.099058 | 0.930581 | 1.298036 | 0.265908 |
| AIM2 | 1.340341 | 1.183437 | 1.518049 | 4.00E-06 |
| ZBP1 | 1.462083 | 1.203033 | 1.776915 | 0.000135 |
| CASP1 | 0.983123 | 0.825602 | 1.170697 | 0.848489 |
| RIPK1 | 0.763385 | 0.598632 | 0.973482 | 0.029508 |
| RIPK3 | 0.994721 | 0.771511 | 1.282508 | 0.967433 |
| MEFV | 1.199855 | 0.966896 | 1.488943 | 0.098066 |
| PYCARD | 1.266538 | 1.082899 | 1.481318 | 0.003112 |
| NLRC4 | 0.93417 | 0.754302 | 1.15693 | 0.532583 |

| Table S3. Drug sensitivity analysis | | | |  |  |  |
| --- | --- | --- | --- | --- | --- | --- |
| Drugs | Low risk group (P value) | High risk group (P value) | | Low risk group (Adj P value) | High risk group Adj P value) |  |
| 5-Fluorouracil |  | 0.0006 | |  | 0.000639 |  |
| Acetalax | 0.00015 |  | | 0.000171 |  |  |
| Afatinib | 1.00E-10 |  | | 3e-10 |  |  |
| AGI-5198 |  | 3.90E-10 | |  | 1.1e-09 |  |
| AGI-6780 | 1.40E-06 |  | | 2.1e-06 |  |  |
| AT13148 | 1.50E-09 |  | | 3.71e-09 |  |  |
| Axitinib | 9.10E-04 |  | | 0.00091 |  |  |
| AZ960 |  | 8.20E-05 | |  | 9.55e-05 |  |
| AZ6102 | 5.60E-12 |  | | 2.05e-11 |  |  |
| AZD1208 | 6.80E-09 |  | | 1.46e-08 |  |  |
| AZD2014 |  | 5.80E-10 | |  | 1.55e-09 |  |
| AZD3759 | 1.40E-12 |  | | 5.33e-12 |  |  |
| AZD4547 | 2.90E-07 |  | | 4.71e-07 |  |  |
| AZD5153 |  | 0.00048 | |  | 4.71e-07 |  |
| AZD5991 | 6.10E-12 |  | | 2.16e-11 |  |  |
| AZD7762 |  | 9.40E-12 | |  | 3.1e-11 |  |
| AZD8055 |  | 3.50E-09 | |  | 8.06e-09 |  |
| BDP-00009066 | 1.90E-06 |  | | 2.81e-06 |  |  |
| BI-2536 | 1.50E-07 |  | | 2.56e-07 |  |  |
| BIBR-1532 | 4.40E-08 |  | | 8.38e-08 |  |  |
| BMS-345541 | 2.22e-16 |  | | 1.37e-15 |  |  |
| Bortezomib | 7.70E-04 |  | | 0.000794 |  |  |
| BPD-00008900 | 6.00E-05 |  | | 7.16e-05 |  |  |
| Camptothecin |  | 2.20E-08 | |  | 4.44e-08 |  |
| Camptothecin | 2.22e-16 |  | | 1.37e-15 |  |  |
| CDK9_5038 |  | 3.50E-08 | |  | 6.93e-08 |  |
| Cediranib | 5.90E-13 |  | | 2.43e-12 |  |  |
| Cisplatin |  | 0.00071 | |  | 0.00074 |  |
| Cyclophosphamide | 8.30E-08 |  | | 1.47e-07 |  |  |
| Dabrafenib |  | 2.10E-11 | |  | 6.5e-11 |  |
| Daporinad | 2.40E-14 |  | | 1.4e-13 |  |  |
| Dihydrorotenone | 2.22e-16 |  | | 1.37e-15 |  |  |
| Elephantin |  | 8.30E-14 | |  | 3.83e-13 |  |
| Entinostat |  | 1.50E-09 | |  | 3.71e-09 |  |
| Epirubicin |  | 1.50E-05 | |  | 1.95e-05 |  |
| ERK_2440 |  | 9.90E-12 | |  | 3.16e-11 |  |
| ERK_6604 |  | 2.22e-16 | |  | 1.37e-15 |  |
| Foretinib |  | 7.60E-10 | |  | 1.98e-09 |  |
| Gallibiscoquinazole | 2.22e-16 |  | | 1.37e-15 |  |  |
| GDC0810 | 5.10E-07 |  | | 8.14e-07 |  |  |
| Gefitinib | 1.70E-07 |  | | 2.85e-07 |  |  |
| Gemcitabine |  | 0.00052 | |  | 0.00056 |  |
| GSK269962A |  | 4.00E-05 | |  | 4.89e-05 |  |
| GSK1904529A | 1.30E-12 |  | | 5.15e-12 |  |  |
| GSK2578215A |  | 9.10E-09 | |  | 1.92e-08 |  |
| GSK2606414 | 0.00014 |  | | 0.000161 |  |  |
| IAP_5620 | 4.40E-08 |  | | 8.38e-08 |  |  |
| I-BRD9 | 4.10E-10 |  | | 1.13e-09 |  |  |
| Ibrutinib | 2.22e-16 |  | | 1.37e-15 |  |  |
| Irinotecan |  | 3.20E-10 | |  | 9.32e-10 |  |
| IWP-2 | 2.70E-05 |  | | 3.38e-05 |  |  |
| KRAS (G12C)Inhibitor-12 | 6.70E-09 |  | 1.46e-08 | |  | |
| LCL161 | 7.80E-14 |  | 3.83e-13 | |  | |
| Leflunomide |  | 0.0004 |  | | 0.000445 | |
| LGK974 | 1.10E-06 |  | 1.68e-06 | |  | |
| LY2109761 | 2.22e-16 |  | 1.37e-15 | |  | |
| MG-132 |  | 1.20E-05 |  | | 1.58e-05 | |
| MIRA-1 | 0.00041 |  | 0.000451 | |  | |
| Mirin |  | 2.22e-16 |  | | 1.37e-15 | |
| Mitoxantrone |  | 4.30E-06 |  | | 6.17e-06 | |
| MK-8776 |  | 4.00E-05 |  | | 4.89e-05 | |
| ML323 | 2.22e-16 |  | 1.37e-15 | |  | |
| MN-64 | 0.00083 |  | 0.000847 | |  | |
| Navitoclax | 7.70E-08 |  | 1.39e-07 | |  | |
| Nelarabine | 1.80E-09 |  | 4.35e-09 | |  | |
| Nilotinib | 1.30E-07 |  | 2.26e-07 | |  | |
| NVP-ADW742 | 4.80E-06 |  | 6.79e-06 | |  | |
| Obatoclax Mesylate |  | 1.20E-08 |  | | 2.47e-08 | |
| OF-1 | 2.22e-16 |  | 1.37e-15 | |  | |
| Osimertinib | 2.22e-16 |  | 1.37e-15 | |  | |
| P22077 | 2.22e-16 |  | 1.37e-15 | |  | |
| PAK_5339 | 5.40E-08 |  | 1.01e-07 | |  | |
| PCI-34051 | 0.00087 |  | 0.000879 | |  | |
| PD173074 | 3.50E-14 |  | 1.93e-13 | |  | |
| PF-4708671 |  | 6.40E-06 |  | | 8.8e-06 | |
| PFI3 | 1.70E-05 |  | 2.19e-05 | |  | |
| Podophyllotoxin bromide |  | 0.00063 |  | | 0.000664 | |
| PRIMA-1MET |  | 2.60E-07 |  | | 4.29e-07 | |
| RO-3306 | 2.22e-16 |  |  | | 1.37e-15 | |
| Ruxolitinib | 3.70E-09 |  | 8.33e-09 | |  | |
| Sabutoclax |  | 8.50E-14 |  | | 3.83e-13 | |
| SB216763 | 2.10E-05 |  | 2.67e-05 | |  | |
| SB505124 | 0.00025 |  | 0.000281 | |  | |
| Selumetinib |  | 3.40E-09 |  | | 8.01e-09 | |
| Sepantronium bromide | 1.20E-05 |  | 1.58e-05 | |  | |
| Sinularin | 2.22e-16 |  | 1.37e-15 | |  | |
| TAF1_5496 | 9.90E-07 |  | 1.53e-06 | |  | |
| Teniposide |  | 1.10E-05 |  | | 1.49e-05 | |
| Topotecan |  | 3.80E-14 |  | | 1.98e-13 | |
| Tozasertib | 7.10E-08 |  | 1.3e-07 | |  | |
| Ulixertinib | 7.40E-07 |  | 1.16e-06 | |  | |
| ULK1_4989 |  | 2.22e-16 |  | | 1.37e-15 | |
| UMI-77 | 2.00E-06 |  | 2.91e-06 | |  | |
| Wee1 Inhibitor | 4.90E-05 |  | 5.92e-05 | |  | |
| WIKI4 |  | 6.50E-05 |  | | 7.66e-05 | |
| Wnt-C59 | 7.80E-12 |  | 2.66e-11 | |  | |
| WZ4003 |  | 6.30E-06 |  | | 8.78e-06 | |
| XAV939 |  | 2.22e-16 |  | | 1.37e-15 | |
| Zoledronate | 1.70E-13 |  | 7.32e-13 | |  | |


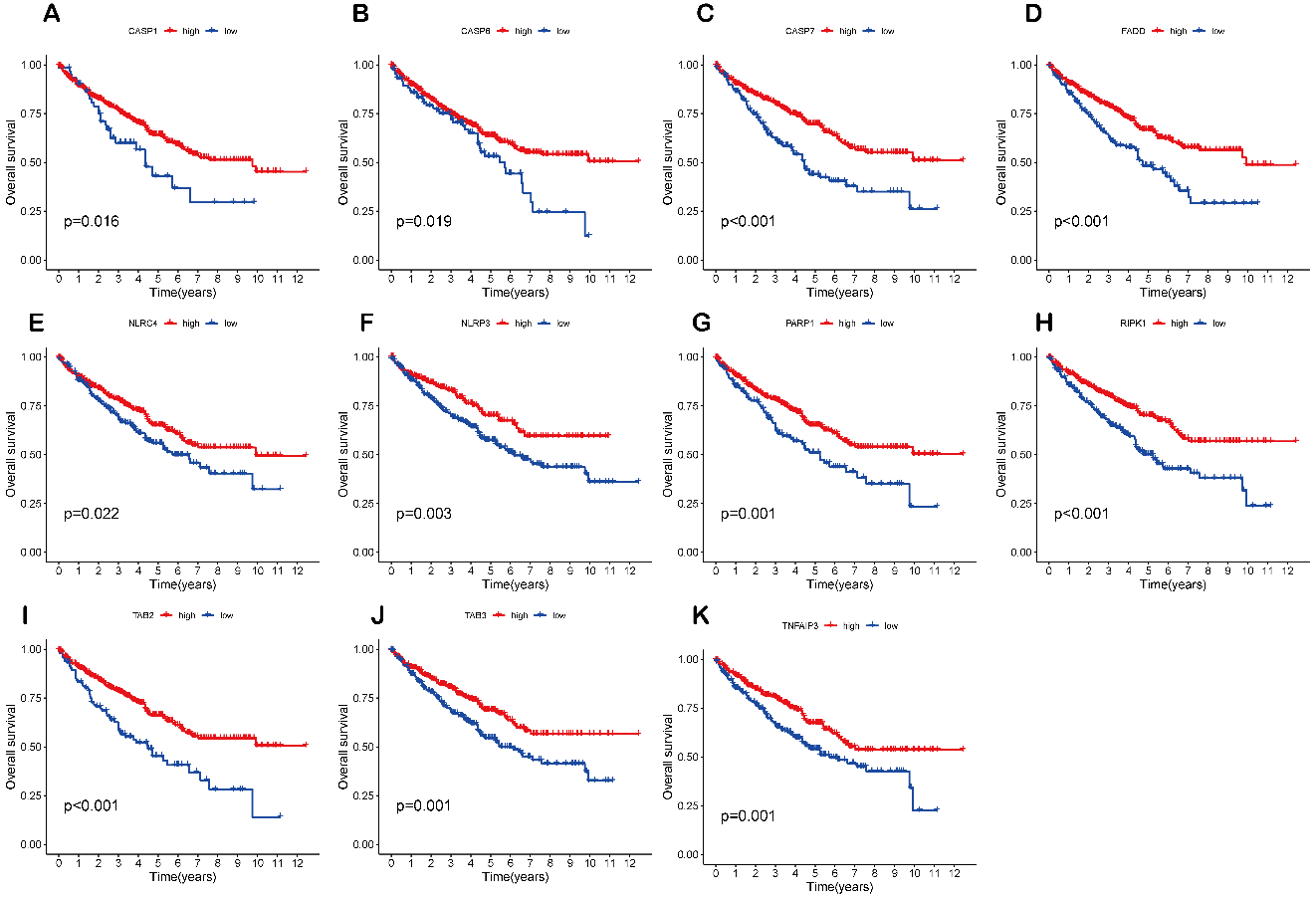


**Figure S1.** Prognosis significance of PANoptosis-related genes of ccRCC patients. (A-K) K-M survival curve displays the OS of ccRCC patients.

**Figure S2.** Evaluation of tumor microenvironment in high- and low-risk groups. (A-K) Relationship between risk score and different immune cell types.


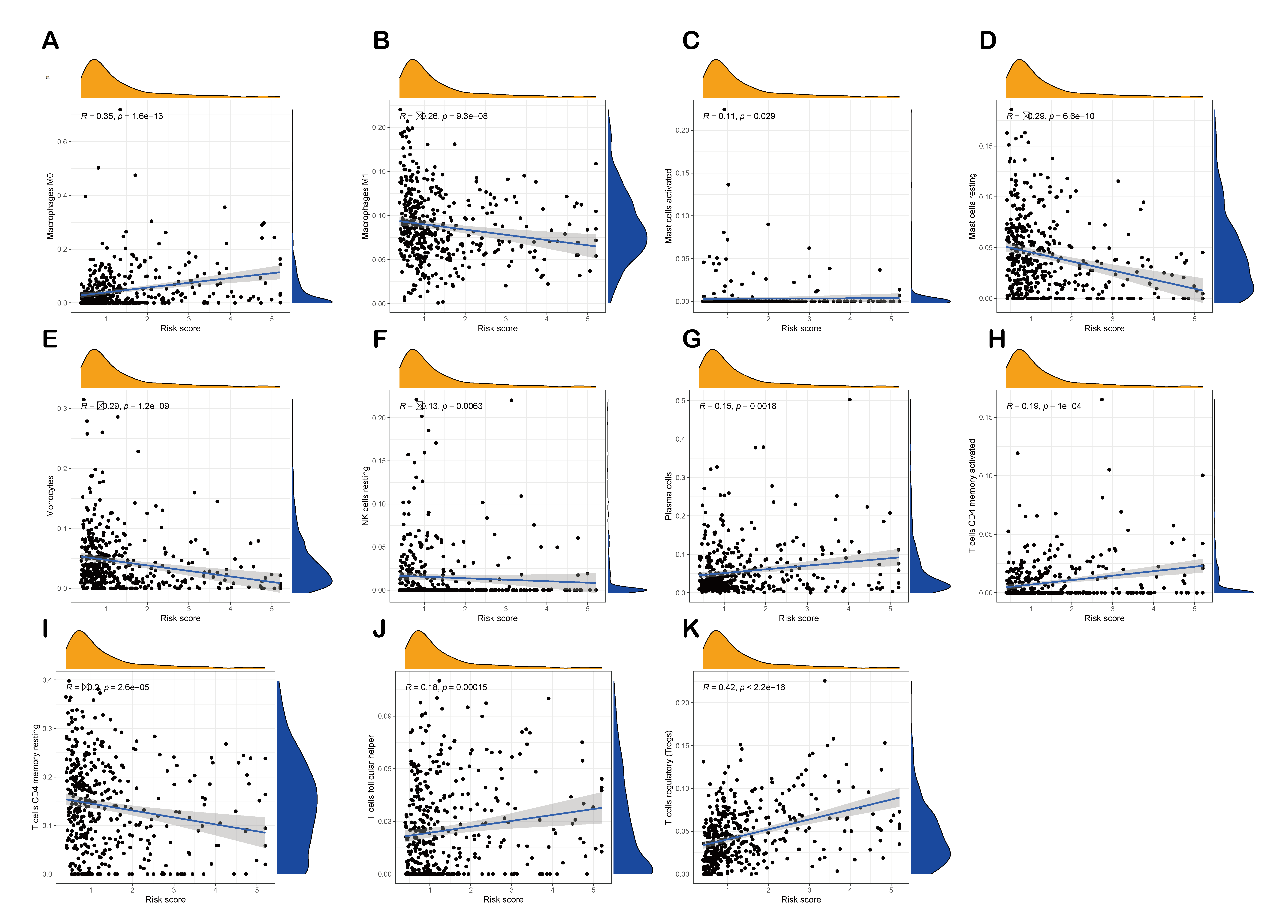

Supplement: Supplementary file 1 [file DataSheet1.docx]
